# Type I IFNs link skin-associated dysbiotic commensal bacteria to pathogenic inflammation and angiogenesis in rosacea

**DOI:** 10.1172/jci.insight.151846

**Published:** 2023-02-22

**Authors:** Alessio Mylonas, Heike C. Hawerkamp, Yichen Wang, Jiaqi Chen, Francesco Messina, Olivier Demaria, Stephan Meller, Bernhard Homey, Jeremy Di Domizio, Lucia Mazzolai, Alain Hovnanian, Michel Gilliet, Curdin Conrad

**Affiliations:** 1 Department of Dermatology, University Hospital CHUV, Lausanne, Switzerland.; 2 Department of Dermatology, Dusseldorf University Hospital, Dusseldorf, Germany.; 3 INSERM UMR 1163, Institut IMAGINE, Necker Hospital for Sick Children, Paris, France.; 4 Department of Angiology, University Hospital CHUV, Lausanne, Switzerland.

**Keywords:** Dermatology, Inflammation, Innate immunity, Mouse models, Skin

## Abstract

Rosacea is a common chronic inflammatory skin disease with a fluctuating course of excessive inflammation and apparent neovascularization. Microbial dysbiosis with a high density of *Bacillus* *oleronius* and increased activity of kallikrein 5, which cleaves cathelicidin antimicrobial peptide, are key pathogenic triggers in rosacea. However, how these events are linked to the disease remains unknown. Here, we show that type I IFNs produced by plasmacytoid DCs represent the pivotal link between dysbiosis, the aberrant immune response, and neovascularization. Compared with other commensal bacteria, *B*. *oleronius* is highly susceptible and preferentially killed by cathelicidin antimicrobial peptides, leading to enhanced generation of complexes with bacterial DNA. These bacterial DNA complexes but not DNA complexes derived from host cells are required for cathelicidin-induced activation of plasmacytoid DCs and type I IFN production. Moreover, kallikrein 5 cleaves cathelicidin into peptides with heightened DNA binding and type I IFN–inducing capacities. In turn, excessive type I IFN expression drives neoangiogenesis via IL-22 induction and upregulation of the IL-22 receptor on endothelial cells. These findings unravel a potentially novel pathomechanism that directly links hallmarks of rosacea to the killing of dysbiotic commensal bacteria with induction of a pathogenic type I IFN–driven and IL-22–mediated angiogenesis.

## Graphical abstract

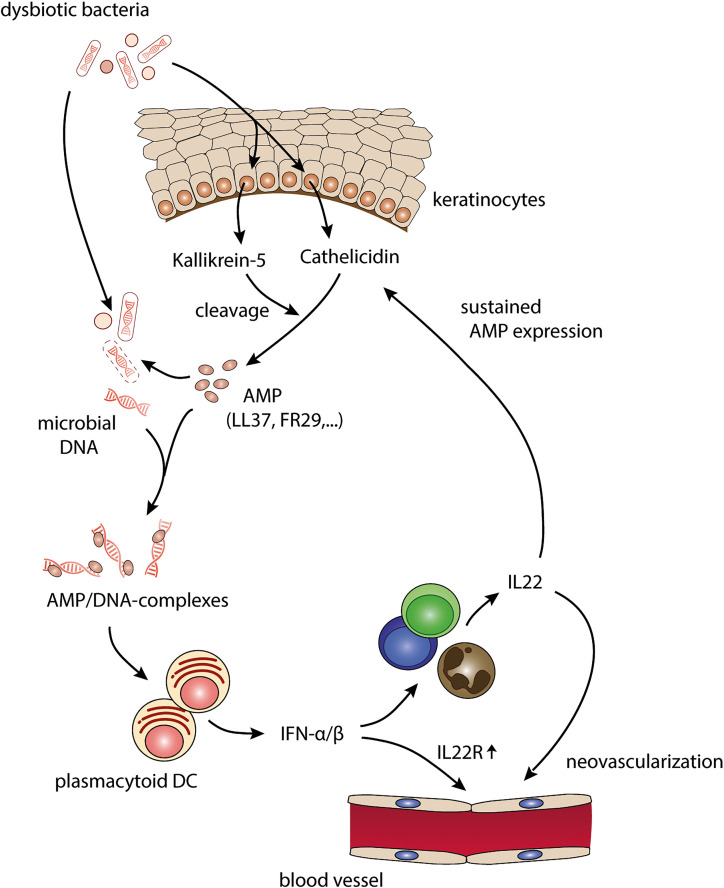

## Introduction

Rosacea is a common chronic inflammatory disorder mainly affecting the centrofacial skin. It is characterized by a variety of clinical manifestations, including transient and persistent erythema, flushing and telangiectasia, papules, pustules, ocular involvement, and, in some cases, phymata. Rosacea often fluctuates between periods of exacerbation and remission. Known trigger factors include temperature changes, UV exposure, emotional stress, exercise, and intake of spicy food or alcohol. More than 5% of the world’s population is affected (1), though the prevalence varies considerably depending on age, sex, geography, and race. Due to its conspicuous nature, rosacea can lead to stigmatization and is often accompanied by depression and anxiety. The psychosocial burden can be debilitating, and rosacea has a significant impact on the emotional, social, and overall well-being of patients (2, 3).

Accumulating evidence suggests a multifactorial etiology with microbial dysbiosis, an aberrant response of the innate immune system, and neurovascular dysregulation playing an important role. Demodex mites, which show a higher density in patients with rosacea (4–6), harbor *Bacillus oleronius*, a commensal bacterium that has been associated with disease activity in rosacea (7–9). The recognition of microbial products by pattern recognition receptors, such as TLR2, which is overexpressed in rosacea (10), induce expression of cathelicidin by keratinocytes (11). Cathelicidin is an antimicrobial peptide (AMP) that necessitates proteolytic processing for activation. Kallikrein 5 (KLK5) represents the predominant protease responsible for cleaving cathelicidin and shows increased expression in rosacea (12). Likewise, both cathelicidin and its processed active forms, including LL-37, FR-29, FA-29, and DI-27, are also overexpressed in lesional skin of patients with rosacea (13). LL-37 has been shown to induce inflammation and edema, drive chemotaxis of various immune cells, and promote angiogenesis, which correlate with characteristics of rosacea (13–15). However, the exact pathogenic role of these AMPs remains to be elucidated for rosacea.

It is well established that LL-37 is able to bind and condense nucleic acids, which are released by dying host cells upon injury, and allows the internalization of these complexes into plasmacytoid DCs (pDCs). Thereby, LL-37 enables activation of endosomal TLR7 and -9 and potent induction of type I IFNs by pDCs (16, 17). Whereas upon skin injury, regulated LL-37 expression leads to transient type I IFN production and protective immune responses (18), overexpression in psoriasis drives excessive pDC activation, resulting in IFN-driven autoimmunity (19–21).

Despite recent scientific advances, the pathomechanisms underlying rosacea are still poorly understood, and current treatment options remain mostly broad and symptomatic. Particularly little is known about early instigators of exacerbations and recurring flare-ups, which would allow development of more targeted, preventive treatment options.

Here, we report that pDC-derived type I IFNs are overexpressed selectively in acute flare-ups of rosacea. In vivo blockade of type I IFN receptor (IFNAR) signaling and pDC depletion both result in a loss of the Th22/Th17 cytokines that are characteristic for rosacea. We show that skin commensal bacteria are necessary and that the rosacea-associated bacterium *B*. *oleronius* is sufficient for the induction of a pathogenic type I IFN–driven immune response by LL-37 in vivo. Moreover, KLK5 cleaves cathelicidin into peptides with heightened DNA binding and type I IFN–inducing capacities, which further facilitate type I IFN production. Finally, type I IFN and IL-22 cooperate in the induction of vascular endothelial cell (EC) survival and proliferation, both in vitro and in vivo, giving rise to aberrant angiogenesis. Thus, several rosacea hallmarks can be directly linked to the killing of dysbiotic commensal bacteria, which initiate type I IFN–driven flare-ups and IL-22–mediated neoangiogenesis, confirming a pathogenic functional role for this pathway in rosacea.

## Results

### Type I IFN signature and Th1/17/22 cytokines, but not Th2 cytokines, are overexpressed in rosacea.

In order to identify potential early instigators of rosacea skin inflammation, we analyzed skin biopsies from patients with rosacea taken either during acute flare-ups or during stabilized chronic inflammation (Supplemental Table 1; supplemental material available online with this article; https://doi.org/10.1172/jci.insight.151846DS1) and compared them with skin from healthy donors.

**Figure 1:**
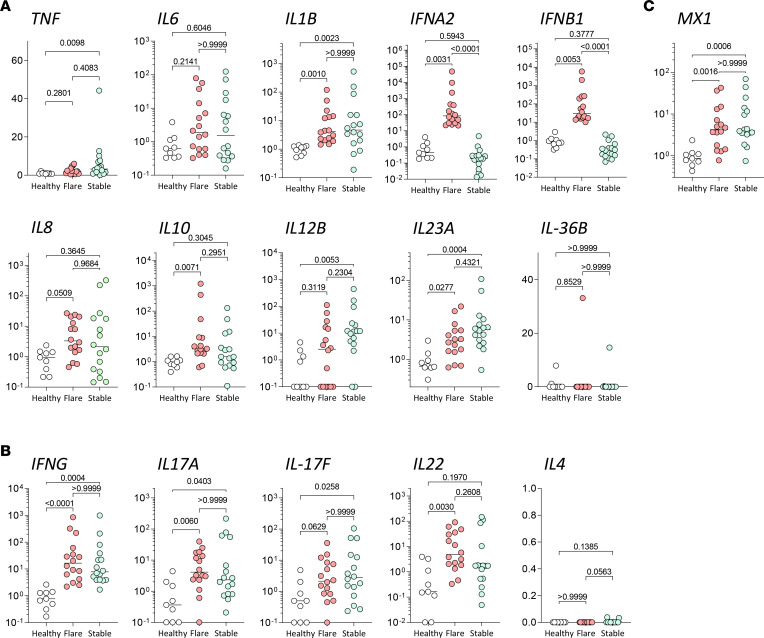
Inflammatory cytokine profile in acute flare-ups and stabilized lesions of rosacea. (**A** and **B**) Gene expression of innate and adaptive inflammatory genes from acute flare-ups (red, *n* = 16) and stabilized lesions (green, *n* = 16) as compared with healthy skin (white, *n* = 9). (**C**) *MX1* expression comparison between healthy skin, flare-ups, and stabilized lesions. Multiplicity adjusted *P* values of 1-way ANOVA with Dunn’s multiple-comparison test are depicted.

First, we confirmed overexpression of the cathelicidin transcript *hCAP18* in rosacea (Supplemental Figure 1A), as described in a previous report (13). We then analyzed mRNA expression levels of selected innate and adaptive cytokines involved in the pathogenesis of different inflammatory skin diseases to identify unique expression patterns. Among innate cytokines, we observed a significant increase in *TNF*, *IL1B*, *IL8*, *IL12B*, *IL23A*, and *IL10* — but not *IL36B* — in rosacea skin lesions as compared with skin from healthy donors (Supplemental Figure 1A and Figure 1A). Furthermore, we could observe a trend increase in *IL6* expression and biphasic distribution for *IFNA2* and *IFNB1*. Adaptive T cell–derived cytokines *IFNG*, *IL17A*, *IL17F*, and *IL22* were significantly overexpressed in lesional rosacea skin, while *IL4* was either undetectable or not increased (Supplemental Figure 1B and Figure 1B). These results are in line with previously published data (22) indicating an inflammation that is predominantly linked to Th1/Th17 cytokine rosacea. When comparing acute flare-ups with stabilized chronic lesions of rosacea in order to identify potentially unique patterns, we found a selective and significant increase in expression of type I IFNs *IFNA2* and *IFNB1* in flare-ups (Figure 1A). Interestingly, whereas type I IFNs were selectively upregulated in patients with rosacea during acute flare-ups, the type I IFN response gene *MX1* was upregulated in all rosacea samples as compared with healthy skin (Supplemental Figure 1C and Figure 1C). Moreover, IHC analysis revealed MxA expression throughout rosacea skin lesions (Supplemental Figure 1, D and E). These findings suggest that type I IFNs are transiently produced early during acute flare-ups of rosacea. All other cytokines revealed comparable expression levels between acute flare-ups and chronic stabilized lesions, though *TNF* was significantly upregulated specifically in more chronic rosacea lesions (Figure 1, A and B).

Taken together, these findings indicate the following: first, rosacea is characterized by predominant Th1/Th17 cytokine signatures, and second, rosacea is driven by early overexpression of type I IFN during acute flare-ups, which then is subsequently relayed by TNF and a chronic inflammation.

### Type I IFN overexpression correlates with pDC infiltration in flare-ups of rosacea.

**Figure 2:**
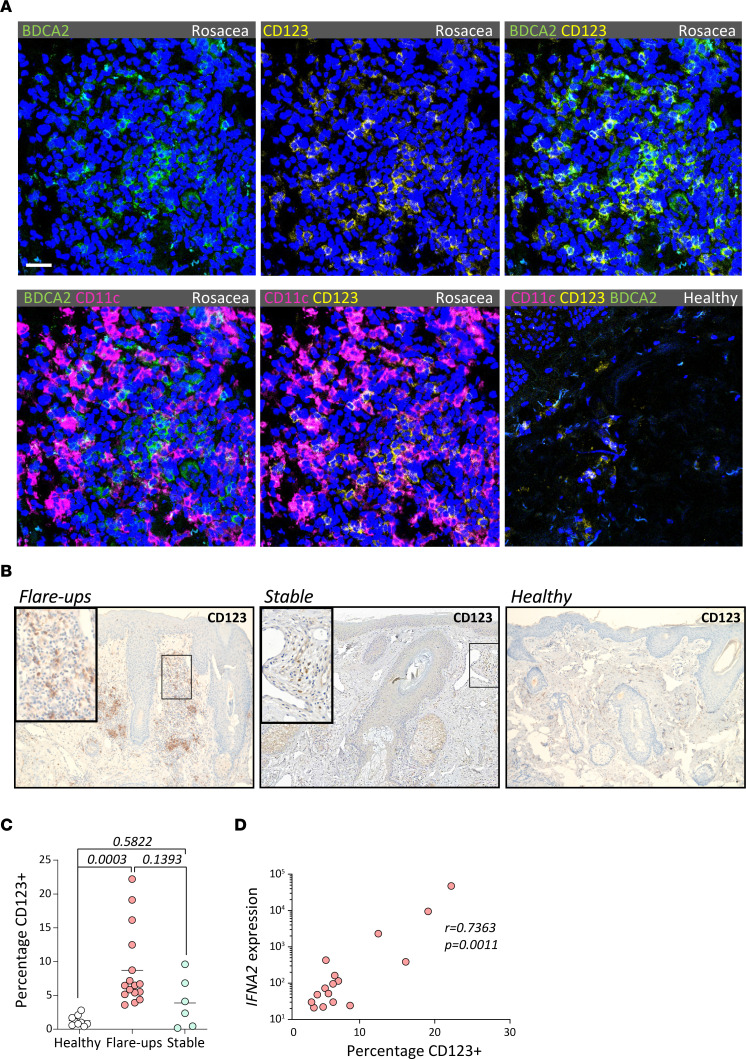
Type I IFN expression correlates with pDC infiltration in acute flare-ups of rosacea. (**A**) Immunofluorescence microscopy of rosacea flare-ups or healthy skin samples costained for BDCA2 (green), CD123 (yellow), CD11c (magenta), and DAPI (blue). Scale bar: 10 μm. (**B**) Rosacea flare-up, stable rosacea, and healthy skin sections stained for CD123^+^ pDCs. Total original magnification, 40×. (**C**) Percentages of CD123^+^ pDCs were determined in relation to nonstromal inflammatory cells per high-magnification field as mean of triplicate measurements per patient sample. (**D**) Percentage CD123^+^ pDCs plotted against IFNA2 expression in lesions from flare-ups. Nonlinear regression with least-squares fit is depicted, with corresponding *P* and *r* values.

As type I IFNs are preferentially expressed by pDCs, we investigated their presence in rosacea by staining paraffin-embedded sections for CD123 (IL-3RA). Lymphoid cells with strong CD123 expression represent bona fide pDCs, as shown by coexpression of BDCA2 and absence of CD11c by microscopy (Figure 2A). While largely absent in facial skin of healthy donors, we found high numbers of pDCs within the dermal infiltrate of rosacea (Figure 2, A and B). Similar to the biphasic expression of type I IFNs, we found significantly more pDCs infiltrating the skin of acute flare-ups but not of chronic stabilized lesions, as compared with healthy skin (Figure 2, B and C). Furthermore, we found statistically significant positive correlation between percentages of CD123^+^ pDCs and both *IFNA2* (Figure 2D; *r* = 0.7363, *P* = 0.0011) and *IFNB1* expression (not shown; *r* = 0.7951, *P* = 0.0002) in patients with acute flare-ups. For patients with stabilized chronic disease, we didn’t find any significant correlation between pDCs and IFNA2 (not shown; *r* = 0.0096, *P* = 0.9856). These findings indicate that pDCs may represent a critical source of type I IFNs during flare-ups of rosacea.

### Type I IFN expression is critically dependent on pDCs in a mouse model of rosacea.

**Figure 3:**
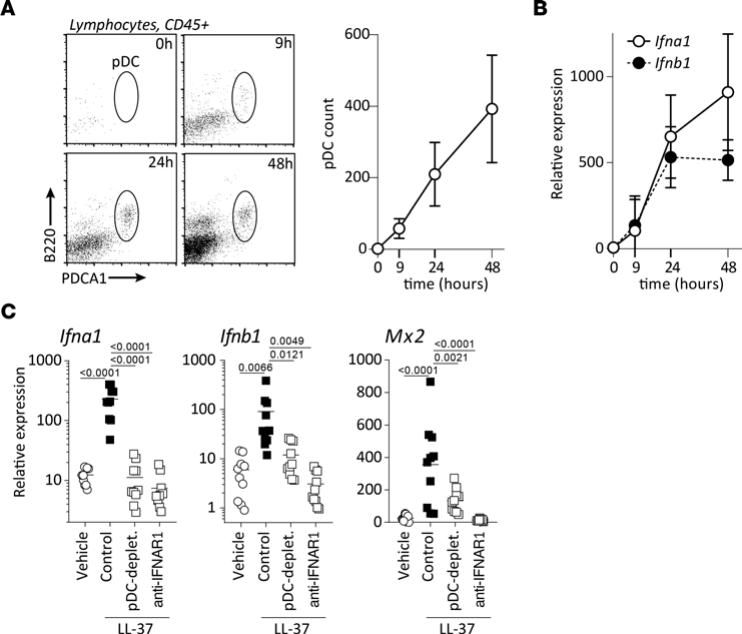
Type I IFN and downstream response gene expression require pDCs in vivo. (**A**) Dot plots from mouse skin biopsies upon LL-37 intradermal injections, pregated on CD45^+^ lymphocytes at the indicated time points and quantification of pDC infiltration. (**B**) Dot plot of *Ifna1* and *Ifnb1* expression within the skin upon LL-37 expression. Values expressed as means ± SD of pooled data from 3 independent experiments. (**C**) Gene expression from biopsies following LL-37 intradermal injection in mice depleted of pDCs or blockaded of type I IFN signaling. Multiplicity adjusted *P* values of 1-way ANOVA are depicted.

To investigate this functionally, we took advantage of a previously described in vivo model of rosacea (13). Briefly, the cathelicidin AMP LL-37, which is overexpressed in skin of patients with rosacea (Supplemental Figure 1) (13), is injected intradermally 4 consecutive times over a period of 48 hours. We found that, upon injection of LL-37, pDCs were rapidly recruited into the skin and accumulate over time (Figure 3A). The pDC accumulation significantly correlated with gene expression of *Ifna1* (*r* = 0.7056, *P* < 0.0001) and *Ifnb1* (*r* = 0.6784, *P* < 0.0001) (Figure 3B). Moreover, depletion of pDCs by anti-Pdca1 antibodies completely abolished *Ifna1* expression and largely abrogated *Ifnb1* expression in the skin (Figure 3C). Congruently, induction of downstream type I IFN–stimulated genes *Mx2* (Figure 3C) and *Ifi202b* (Supplemental Figure 2A) were significantly reduced — in fact, entirely abolished — for *Isg15* and *Irf7* (Supplemental Figure 2A). We obtained similar results using a different pDC depletion system, the BDCA2-DTR transgenic mouse (Supplemental Figure 2B), confirming in vivo that pDCs are the principal source of type I IFNs.

### Type I IFN blockade and pDC depletion abolish select Th1/Th22 signatures.

**Figure 4:**
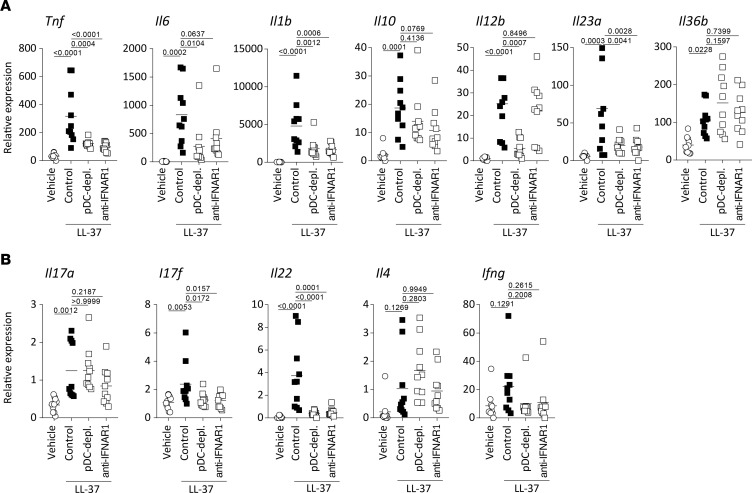
Type I IFN and pDC contribute to Th1- and Th22- but not Th2-related inflammatory cytokine expression in situ. (**A** and **B**) Innate and adaptive cytokine expression in biopsies obtained from intradermally injected mice with corresponding pre-treatments. Multiplicity adjusted *P* values of 1-way ANOVA are depicted.

Next, we sought to analyze the functional role of pDC-derived type I IFNs in driving pathogenic inflammation in rosacea. First, we investigated whether the in situ cytokine expression profile in the mouse model indeed reflects human rosacea. The expression of proinflammatory innate cytokines, including Th1 polarizing cytokines *Il12b* and *Tnf*; Th17/Th22 polarizing cytokines *Il1b*, *Il23a*, and *Il6*; and *Il10* were significantly induced as compared with controls showing a similar expression profile as in rosacea (Figure 4A). Overexpression of *Tnf*, *Il1b*, *Il23a*, and *Il6* was critically dependent on pDCs and type I IFN signaling, while induction of *Il36b* and *Il10* expression was independent of either. Among adaptive cytokines tested, Th17/Th22 cytokines *Il17a*, *Il17f*, and *Il22*, but not *Il4* and *Ifng*, were significantly induced upon LL-37 injection. However, only the overexpression of *Il17f* and *Il22* was dependent on pDCs and type I IFN signaling (Figure 4B). These findings indicate that LL-37 overexpression in rosacea activates skin-infiltrating pDCs to produce type I IFN, which is required to drive a skin inflammation with increased expression of Th17/Th22 cytokines.

### Cathelicidin peptides found in rosacea display differential potencies to bind and complex DNA and to induce type I IFNs.

**Figure 5:**
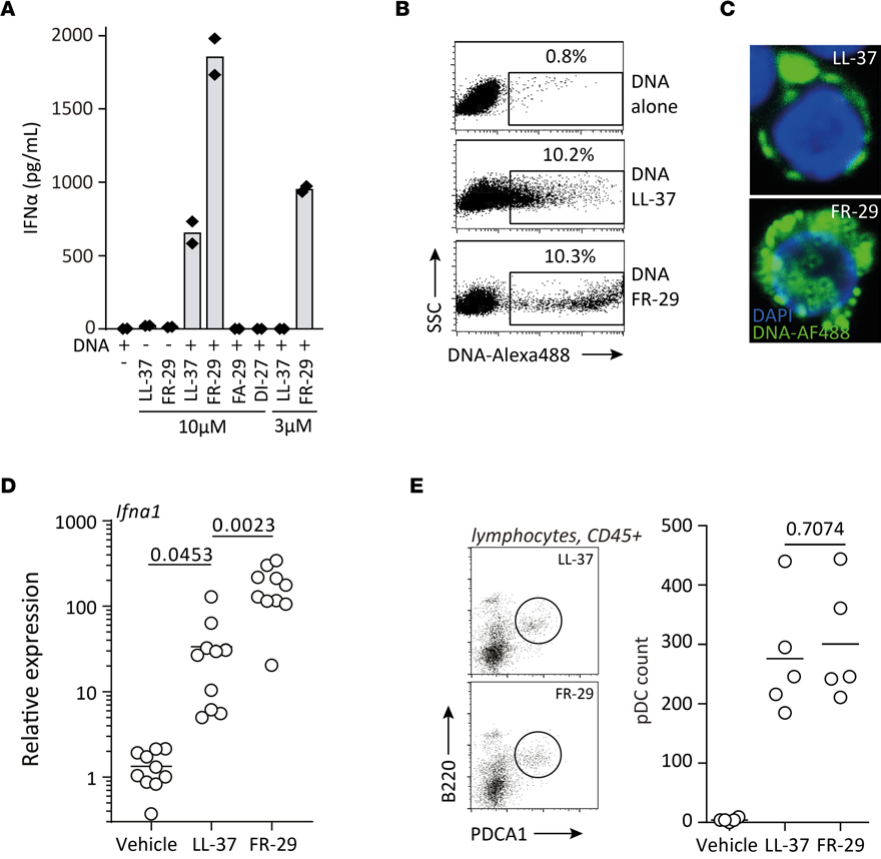
Cathelicidin peptide FR-29 activates pDCs more potently than LL-37 by binding DNA with more affinity, resulting in more potent internalization of nucleic acids. (**A**) Isolated human pDCs were stimulated with the indicated cathelicidin peptides complexed with DNA, and IFN-α was measured from supernatants after a 24-hour stimulation. (**B**) DNA was labeled with Alexa Fluor 488 according to manufacturer’s instructions, incubated with the indicated cathelicidin peptide, and put in culture with isolated human pDCs for 3 hours before measuring the fluorescence by flow cytometry. (**C**) Cytospin of pDCs stimulated in the presence of fluorescently labeled DNA and the indicated peptide. Total original magnification, 400×. (**D**) LL-37, FR-29, or vehicle control (saline) were injected intradermally into WT Balb/c mice, and biopsies were taken for gene expression analysis. (**E**) pDC quantification by flow cytometry. Multiplicity adjusted *P* values of 1-way ANOVA with Dunnett’s multiple-comparison test are depicted in **D**, and *P* value of unpaired *t* test with Welch’s correction is depicted in **E**.

It is established that LL-37 is able to activate pDCs to produce large amounts of type I IFNs by internalizing extracellular nucleic acids (16). In rosacea, however, cathelicidin is processed into several additional C-terminal peptides besides LL-37, such as FR-29, FA-29, and DI-27 (Supplemental Figure 3) (13). These, despite considerable overlap (Supplemental Figure 3B), display significant differences in terms of hydrophobic surfaces (Supplemental Figure 3, A–C), charge, and percentage of predicted α-helical structures (Supplemental Figure 3D). Thus, we wondered whether these peptides had a differential ability to activate pDCs. We isolated human pDCs and stimulated them with complexes of DNA and the different cathelicidin-derived peptides. When complexed with DNA, FR-29 showed significantly increased IFN-α production by pDCs as compared with LL-37 for the same molar concentration (Figure 5A). Neither FA-29 nor DI-27 were able to stimulate detectable amounts of IFN-α from pDCs in vitro. When reducing the concentration of peptides below the stimulatory capability of LL-37 to 3 μM, FR-29 retained significant — albeit reduced — stimulatory capacity. To analyze the underlying mechanism of differential IFN-α production, we first assessed the DNA binding capacity of these peptides by picogreen fluorescence quenching. Indeed, FR-29 bound and condensed DNA more potently than LL-37 (Supplemental Figure 4A). Conversely, FA-29 and DI-27 had almost indiscernible DNA binding activity. To further assess the capacity of FR-29 and LL-37 to form DNA-peptide complexes and to mediate internalization of DNA into pDCs, we fluorescently tagged purified, undigested DNA and complexed it with the cathelicidin peptides LL-37 and FR-29. Whereas pDCs were unable to uptake DNA in the absence of cathelicidin peptides, LL-37 and FR-29 both caused uptake of fluorescently labeled DNA by approximately 10% of cells (Figure 5B). However, the relative uptake of DNA was markedly more prominent when complexed with FR-29 than with LL-37, as shown by the increased fluorescence intensity (Figure 5, B and C). Taken together, these data indicate that several fragments found in rosacea, such as FA-29 and DI-27 cathelicidin peptides, fail to activate pDCs, whereas FR-29 has a significantly more potent effect than LL-37 due to increased affinity to nucleic acids.

To further evaluate the in vivo relevance of these findings, we analyzed *Ifna* gene expression in the rosacea mouse model. Indeed, intradermal injection of FR-29 induced significantly more potent *Ifna1* production than LL-37 (Figure 5D). pDC infiltration of the skin was comparable between FR-29 and LL-37, suggesting that enhanced *Ifna1* production upon FR-29 injection is due to a differential activatory capacity of the cathelicidin peptides rather than increased skin infiltration by pDCs (Figure 5E).

### KLK5 recapitulates pDC-dependent type I IFN expression.

**Figure 6:**
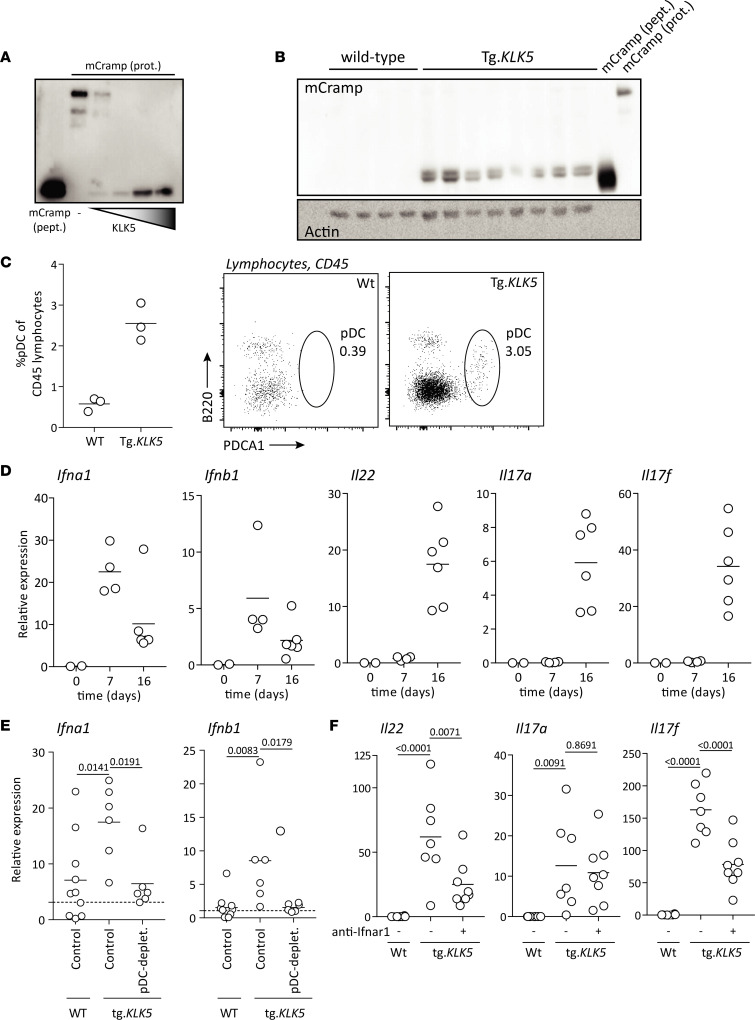
Kallikrein 5 overexpression in the epidermis results in pDC-derived overexpression of type I IFN and IFN-sustained IL-22 expression. (**A**) Murine Cramp recombinant protein (mCramp prot.) incubated with human KLK5 recombinant protein (hKLK5) for increasing times (5, 15, 30, and 90 minutes). Synthetic murine Cramp peptide (mCramp pept.) is used as a control for staining. (**B**) Skin samples from transgenic *KLK5* mice (Tg.*KLK5*) and WT littermates was homogenized and probed for mCramp by Western blot. Synthetic mCramp peptide and mCramp protein was used as a control for staining. (**C**) Skin lesions from 7-day-old K5.*KLK5* transgenic mice and the corresponding skin region from age-matched WT littermates was stained for pDCs by flow cytometry. (**D**) Gene expression from newborn, 7-, and 16-day-old transgenic K5.*KLK5* mice. (**E**) Transgenic K5.*KLK5* mice and WT littermates were depleted of pDCs from birth by s.c. injection of depleting antibodies, or injected with saline, every 2 days. Skin gene expression was assessed. (**F**) Transgenic K5.*KLK5* and WT littermates were treated with anti-Ifnar antibodies, or injected with saline, every 2 days. Skin gene expression assessed. Multiplicity adjusted *P* values of 1-way ANOVA with Dunnett’s multiple-comparison test are depicted.

Cathelicidin propeptide processing is required for LL-37 activity in the skin. KLK5, which is overexpressed in rosacea (10, 13), mediates processing of cathelicidin into the active LL-37 peptide. To determine whether KLK5 overexpression is sufficient to instigate activation of the LL-37/pDC/type I IFN axis, we took advantage of a recently described transgenic murine model expressing human KLK5 in the epidermis under the K5 promoter (Tg.*KLK5*) (23). First, we confirmed in vitro and in vivo that human KLK5 is able to cleave Cramp propeptide, the murine analogue of human cathelicidin. In vitro*,* human KLK5 effectively processed murine Cramp propeptide, as shown by Western blot (Figure 6A). In addition, spontaneously after birth, we found significantly increased expression of *Cramp* propeptide and increased presence of the cleaved active form of Cramp in the skin of Tg.*KLK5* mice, as compared with WT littermates (Figure 6B). These findings indicate that constitutive overexpression of KLK5 in the skin leads to accumulation of active cathelicidin peptides similar to those seen in rosacea. Next, we investigated the presence of pDCs in skin of these mice. Seven days after birth, pDCs accumulated significantly and spontaneously in Tg.*KLK5* mice but not control mice (Figure 6C) and were paralleled by type I IFN overexpression (Figure 6D). Whereas type I IFNs were upregulated early in the skin and declined over time, Th17-related cytokines *Il22*, *Il17a*, and *Il17f* were significantly overexpressed only at a later time point, indicating that type I IFNs might drive a Th17 cytokine–mediated response in Tg.*KLK5* mice. Depletion of pDCs from birth led to loss of induction of type I IFN in the skin, confirming pDCs as the principal sources of type I IFN in the Tg.*KLK5* mouse model (Figure 6E). Furthermore, blockade of type I IFN signaling significantly inhibited expression of Th17/Th22 cytokines *Il22* and *Il17f*, in line with data obtained in the LL-37 intradermal injection model (Figure 6F). Taken together, these findings indicate that KLK5 overexpression in rosacea increases cleavage of cathelicidin in the skin. In turn, accumulating active cathelicidin peptides attract and activate pDCs, resulting in a type I IFN–driven immune response with increased expression of Th17/Th22 cytokines, which is characteristic for rosacea.

### Cathelicidin peptides kill bacteria associated with rosacea, leading to activation of pDCs.

**Figure 7:**
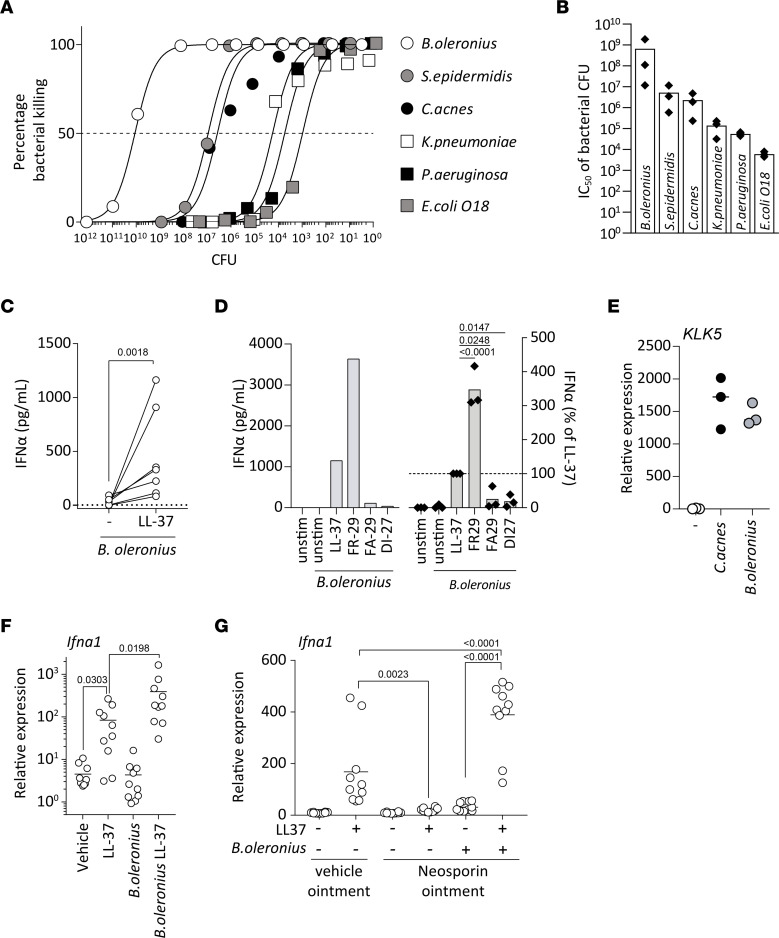
Cathelicidin-mediated killing of rosacea-associated bacteria activates pDCs to produce IFN-α. (**A**) Bacteria at the indicated CFU were incubated with LL-37 at a constant 10 μM concentration for 3 hours; subsequently, CFUs were counted after 18 hours of culture in their appropriate culture conditions following serial dilution. (**B**) CFU numbers efficiently killed with a constant concentration of LL-37 as in **A** and expressed as calculated IC_50_ values for each of the indicated bacteria. (**C**) Plasmacytoid DCs were isolated from human blood and stimulated with live *B*. *oleronius*, or *B*. *oleronius* preincubated with either LL-37 or left unstimulated (dotted line) for 24 hours, and IFN-α production was assessed by ELISA. Results are pooled from 7 donors. (**D**) pDCs were stimulated as in **C** with either *B*. *oleronius* alone or in preincubated with 5 μM of the indicated cathelicidin peptide. Representative data from a single donor (left) and of at least 3 donors per condition expressed as percentage of each donors’ LL-37 + *B*. *oleronius* condition. *P* values of 2-tailed paired *t* test are depicted. (**E**) Normal human epidermal keratinocytes were stimulated with 1 × 10^3^ CFU of *B*. *oleronius* or *C*. *acnes*, or left unstimulated, and gene expression changes were quantified by qPCR. (**F**) IFN-α gene expression from biopsies collected from mice injected with LL-37, heat-killed *B*. *oleronius*, or heat-killed *B*. *oleronius* preincubated with LL-37 over the course of 24 hours. (**G**) Neosporin- (containing Neomycin, Bacitracin, Polymyxin B) or vehicle ointment–treated mice were injected intradermally over the course of 48 hours with the indicated combinations of saline, LL-37, or LL-37 preincubated with *B*. *oleronius*. *P* values of 2-tailed paired *t* test are depicted in **C**. Multiplicity adjusted *P* values of 1-way ANOVA are depicted in **D**, **F**, and **G**.

It has been shown that complexes of host DNA and AMPs such as LL-37 play an important role in the pathogenesis of several chronic inflammatory diseases such as psoriasis, lupus erythematosus, or atherosclerosis (16, 24, 25). Because rosacea severity has been linked to dysbiotic communities of commensal skin bacteria — in particular, *B. oleronius* and *Staphylococcus epidermidis —* we wondered whether commensal microbial DNA rather than host DNA could play a pathogenic role in driving inflammation in rosacea. First, we investigated killing of different skin- and non–skin-associated bacteria by LL-37. *B*. *oleronius* was the most susceptible, followed by the other skin-associated bacteria *S*. *epidermidis* and *Cutibacterium acnes* (Figure 7, A and B). Gut- and lung-associated bacteria, though still subject to killing, were far less susceptible, indicating a preferential killing of skin commensals. When comparing the different rosacea-associated cathelicidin peptides, we found that FR-29 and FA-29, but not DI-27, were able to kill all bacteria tested (Supplemental Figure 4, B and C). Similar to its ability to condense DNA and activate pDCs, FR-29 showed a higher potency than LL-37 in killing *B*. *oleronius* and *S*. *epidermidis*, while the latter was slightly more potent or at least equally efficient in killing the other strains of bacteria tested (Supplemental Figure 4, B and C). These data indicate that the different cathelicidin peptides found in the skin of patients with rosacea show a slightly different spectrum of antimicrobial activity. Moreover, FA-29, which is not able to bind and condense DNA, efficiently killed several strains of bacteria at similar concentrations as LL-37. Thus, the ability of cathelicidin peptides to condense DNA and activate endosomal TLRs is not directly linked to their antimicrobial capacity to kill bacteria.

Next, we tested if LL-37–killed *B*. *oleronius* could serve as a DNA source and activate pDCs in vitro. We isolated human pDCs from healthy donors and cultured them in the presence of live bacteria with or without LL-37. Whereas *B*. *oleronius* alone was not able to activate pDCs, the addition of LL-37 allowed for potent activation of pDCs, leading to important production of IFN-α (Figure 7C). In accordance with our previous results, FR-29 was able to induce significantly more IFN-α by pDCs than LL-37 when in the presence of *B*. *oleronius*, whereas FA-29 and DI-27 displayed a poor capacity to activate pDCs (Figure 7D).

Since *C. acnes* has previously been shown to upregulate KLK5 expression on keratinocytes through TLR2 (10), we tested to discover whether *B*. *oleronius* can also induce KLK5 expression. Both bacteria showed a similar capacity to induce KLK5 in vitro (Figure 7E), indicating that *B*. *oleronius* not only provides an essential source for DNA, but also induces cleavage of cathelicidin through upregulation of KLK5 in order to activate pDCs and induce type I IFN production in flare-ups of rosacea.

To further investigate the in vivo relevance of these findings, we took advantage of the rosacea mouse model and injected heat-killed *B*. *oleronius* alone or preincubated with LL-37. Interestingly, *B*. *oleronius* alone was not able to induce type I IFN expression within 24 hours. Preincubated with LL-37, however, *B*. *oleronius* induced significantly more type I IFN expression than LL-37 alone (Figure 7F). These findings indicate that an increased bacterial load alone is not sufficient to rapidly engage the type I IFN pathway but, instead, that bacterial killing by cathelicidin peptides is required. Since *B*. *oleronius* further increased type I IFN expression within the skin upon LL-37 injections, we wondered whether commensal skin bacteria are actually a prerequisite to activate pDCs and induce IFN-α production in our rosacea model. Thus, we pretreated mice with topically applied wide-spectrum antibiotics for 2 days, prior to LL-37 injections. We found that type I IFN expression was almost completely abolished upon antibiotic treatment of the skin (Figure 7G), whereas injection of *B*. *oleronius* with LL-37 was sufficient to restore and enhance type I IFN even further.

Thus, skin commensal bacteria are required but, by themselves, not sufficient to rapidly activate pDCs and induce type I IFN in rosacea. On the other hand, *B*. *oleronius* is sufficient for cathelicidin-driven overexpression of type I IFN in the skin. Taken together, these findings indicate a functional role for dysbiotic communities of commensal bacteria in triggering a pathogenic immune response and flare-ups of rosacea.

### Type I IFN and IL-22 cooperate in the induction of pathogenic angiogenesis.

**Figure 8:**
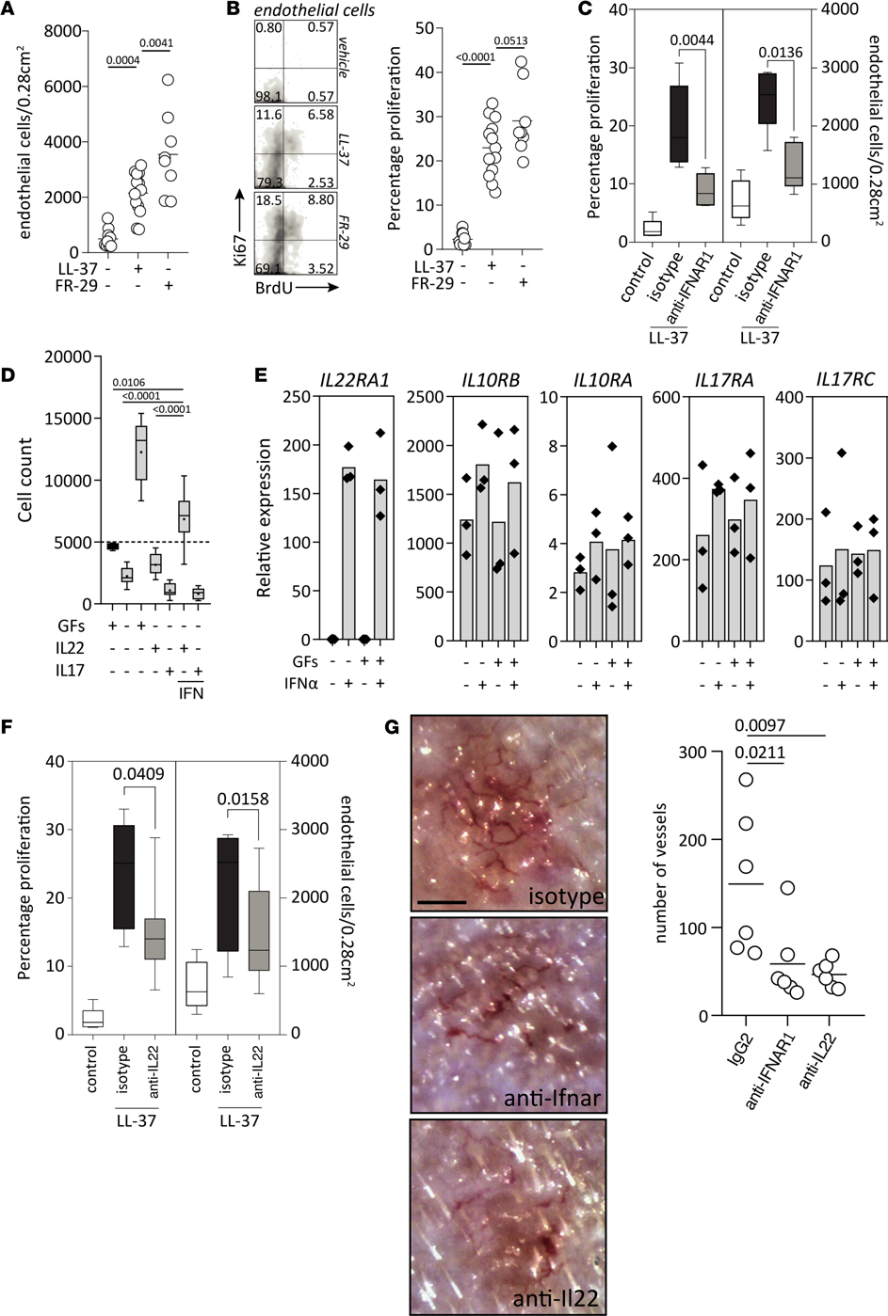
Type I IFN cooperates with IL-22 in the induction of angiogenesis by promoting EC survival and proliferation. (**A**) The indicated cathelicidin peptides, or saline, were injected intradermally 4 times over a 48-hour period as previously, followed by intraperitoneal injection with BrdU for 72 hours. EC numbers were defined as Cd31^+^Cd45^–^ cells from debris-excluded pregating, and they were determined per injection site. (**B**) Proliferation was determined by staining of Ki67 and BrdU from cells gated on ECs as in **A**, and defined as Ki67–single positive, Ki67-BrdU–double positive, and BrdU–single positive cells. (**C**) Mice intradermally injected with LL-37 or saline as in **A** and **B** were either pretreated with an anti-Ifnar blocking antibody or an IgG2a. EC numbers and proliferation were determined as in **A** and **B**. (**D**) HUVECs were plated (black bar at time of plating) and kept with or without growth factors (GFs) EGF, IGF1, FGF2, and VEGFA and with or without IL-22 or IL-17, in the presence or absence of IFN-α. Live cells were defined as CytoGreen^+^CytoXOrange^–^ cells and depicted as absolute counts. (**E**) HUVEC were stimulated for 8 hours in the indicated conditions, and gene expression was analyzed for the designated genes. Results are representative of 3 independent experiments. (**F**) Mice were treated as in **C** and pretreated with an anti–IL-22 blocking antibody or an IgG2a, and EC counts and proliferation were assessed as in **C**. (**G**) Mice were treated as in **C**, with either an IgG2a, anti-Ifnar, or anti–IL-22 antibody, and vessels were visualized by videocapillaroscopy and counted in and around the injection site. Scale bar: 250 μm. Multiplicity adjusted *P* values of 1-way ANOVA are depicted in **A**, **B**, **D**, and **G**. *P* values of 2-tailed Mann-Whitney nonparametric unpaired *t* test are depicted in **C** and **F**.

Hallmarks of rosacea include recurrent episodes of flushing and, eventually, fixed centrofacial erythema, which is caused by neoangiogenesis and telangiectasia. Therefore, we wondered whether the KLK5–LL-37‒pDC‒IFN-α‒Th17/Th22 axis could play a pathogenic role in driving neoangiogenesis in rosacea. First, we assessed the number of dermal ECs as well as their proliferative status in the rosacea mouse model. LL-37 injections significantly increased the number of ECs (Figure 8A) and the percentage of proliferating ECs (Figure 8B) as compared with control injections. Since FR-29 injections further augmented numbers and proliferation of ECs, we questioned whether the proangiogenic effect of cathelicidin peptides (14) might be through IFN-α–driven induction of Th17/Th22 cytokines. Indeed, blockade of type I IFN signaling significantly reduced both the overall numbers and percentage of proliferating ECs (Figure 8C). To further dissect the mechanism, we sought to determine the effect of IFN-α on primary ECs, which are dependent on growth factors for survival and proliferation in culture. While EC growth factors maintained viability and markedly increased cells numbers in vitro, IFN-α did not have any discernible direct effect on EC viability and proliferation (Supplemental Figure 5, A–C). Therefore, we tested if type I IFN–induced downstream Th17/Th22 cytokines were instead able to promote EC proliferation. Neither IL-17 nor IL-22 alone had a direct effect on EC viability. However, ECs cultured in the presence of both IFN-α and IL-22 showed maintained viability and significantly increased cell numbers. Interestingly, this effect was specific for IL-22, since IL-17A did not augment EC viability when combined with IFN-α (Figure 8D and Supplemental Figure 5, C and D). Since IFN-α is required for IL-22–driven EC proliferation, we wondered if IFN-α mediated its effect via upregulation of the IL-22 receptor on ECs. The expression of IL-22RA1 — the receptor subunit specific for IL-22 — was absent on unstimulated ECs but greatly and significantly induced upon stimulation with IFN-α (Figure 8E). On the other hand, IL-17RA and IL-17RB — which form the active receptor for IL-17 as well as IL-10RB, the second subunit of the IL-22 receptor — were constitutively expressed and not altered by addition of IFN-α or growth factors. These results show that IFN-α renders ECs susceptible to IL-22 through upregulation of its cognate receptor, thereby enabling IL-22–driven proliferation. To verify this in vivo, we assessed EC numbers and proliferation upon IL-22 blockade in the rosacea mouse model (Figure 8F). Indeed, inhibition of IL-22 significantly reduced the numbers and percentage of proliferating ECs in the skin (Figure 8F), similar to type I IFN blockade (Figure 8C). Histopathological evaluation showed that inhibition of IL-22 and blockade of type I IFN signaling both markedly reduced the inflammatory infiltrate in the skin but did not abolish it entirely (Supplemental Figure 6). LL-37 injections led to neoangiogenesis and formation of visible microvessels, as assessed by in vivo videocapillaroscopy, which resembled images taken from patients with rosacea (Supplemental Figure 5E). These microvessels were significantly reduced upon IFNAR blockade and inhibition of IL-22 (Figure 8G). In summary, overexpression of cathelicidins induces vascular EC proliferation and neoangiogenesis in rosacea via induction of pDC-derived type I IFN, which in turns leads to upregulation of IL-22 and expression of the IL-22 receptor on ECs, thereby enabling IL-22–driven neoangiogenesis.

## Discussion

Our study identifies key pathogenic mechanisms driving acute flare-ups of rosacea. We found early overexpression of type I IFN in acute rosacea correlating with accumulating pDCs in the dermal infiltrate of rosacea skin lesions. Using both in vitro and in vivo models, we demonstrate that commensal skin bacteria are necessary for pDC activation and type I IFN production, which, in rosacea, is further amplified by dysbiotic bacteria and AMPs with increased type I IFN–inducing capacities. Type I IFNs then drive a pathogenic immune response with increased IL-22 expression and simultaneously render ECs susceptible to IL-22 through upregulation of its receptor. Thereby, type I IFNs link skin dysbiosis to neoangiogenesis, one of the pathological hallmarks of rosacea.

Previous studies have demonstrated that KLK5 and cathelicidin peptides have a functional role in driving an inflammatory response in rosacea. Here, we unravel the underlying pathomechanisms. KLK5, which is overexpressed in rosacea, cleaves cathelicidin into different peptides. The cathelicidin peptides lead to infiltration of the skin by pDCs, which in turn are activated by complexes of these cathelicidin peptides and nucleic acid to produce large amounts of type I IFNs. In a self-amplifying loop, type I IFNs then attract, through induction of CXCR3 ligands (26–28), additional pDCs to the skin. The resulting type I IFN overexpression eventually induces the strong immune response with increased expression of Th22/Th17 cytokines that is characteristic for rosacea.

Though the exact mechanisms are currently unclear, type I IFNs have been shown to drive Th17-type inflammatory responses (18, 29). Type I IFNs can initiate T cell responses by inducing differentiation and maturation of conventional DCs (30). They may more specifically promote Th22/Th17 immune responses by differentiating monocytes into DCs that produce IL-23 (31). Furthermore, type I IFNs condition pDCs to become potent drivers of Th17 polarization (32). Predominant accumulation of Th1 and Th17 cells in the dermal infiltrate of rosacea has indeed been shown (22). However, the functional relevance of T cells in the pathogenesis of rosacea is still unclear, and the cellular source of the pathogenic Th17/Th22 cytokines remains elusive. Besides T cells, potential candidates include type 3 innate lymphoid cells (ILC3) and NK cells, mast cells, and neutrophils, which have all been reported to express IL-17 and/or IL-22 (33–37). Moreover, Th17 cells, mast cells, and neutrophils have been shown to accumulate in rosacea (22).

We found that pDCs represent the principal cellular source of IFN-α– and — for the most part — IFN-β–production in vivo. However, some residual IFN-β and IFN-response gene expression are discernible, despite pDC-depletion, and skin of chronic stabilized rosacea shows a diffuse MxA-staining pattern. Thus, other cell types might contribute to the type I IFN production in rosacea. Keratinocytes (38–40) and ECs (41) have indeed been shown to produce IFN-β in response to sensing nucleic acids complexed with LL-37 via endosomal or cytosolic receptors.

Self-DNA and self-RNA complexed with AMPs are able to activate endosomal TLRs of pDCs (16, 17). However, we show here that commensal skin bacteria are required as a source of nucleic acid in order to induce relevant pDC activation and type I IFN production in vivo, in line with similar observations from skin wound healing (29). These findings suggest that bacteria have several key pathogenic roles in rosacea: they (a) activate TLR2 on KCs to upregulate KLK5 (10), (b) provide an essential source for nucleic acid in order to activate pDCs to produce type I IFN and initiate a pathogenic inflammation, and (c) activate neutrophils through cell-surface TLRs (8). Therefore, bacteria are essential for the activation of the KLK5–cathelicidin–pDC/type I IFN axis.

We found that the skin commensal *B*. *oleronius,* previously shown to be dysbiotic in rosacea (9), further amplifies type I IFN production. In fact, compared with other commensal bacteria — in particular, compared with *C*. *acnes*, which plays an important pathogenic role in acne, a disease with strong clinical resemblance to rosacea — *B*. *oleronius* is highly susceptible and preferentially killed by cathelicidin AMPs. This leads to enhanced generation of complexes with DNA facilitating the activation of pDCs. The importance of dysbiotic communities of commensal bacteria in the pathogenesis of rosacea is further supported by the fact that commensal *C*. *acnes* shows lower relative abundance in patients with rosacea and is further reduced with increased disease severity (42, 43). On the other hand, *B*. *oleronius* has been linked to disease activity in rosacea (8, 44). Demodex mites, which harbor *B*. *oleronius*, are markedly increased in patients with rosacea as compared with healthy controls (45). In fact, Demodex mites are found within the pilosebaceous unit, which is often at the epicenter of the inflammatory infiltrate in papulopustular rosacea. Demodex can directly activate TLR2 and, therefore, contribute themselves to the induction of an inflammatory response in rosacea. Nevertheless, papulopustular rosacea improves upon antibiotic treatments, which do not display any activity against Demodex. It has been suggested that the clinical efficacy of tetracycline is based on its bystander antiinflammatory properties. However, antiinflammatory treatments, such as topical steroids and tacrolimus, often aggravate or even induce rosacea (46, 47), indicating that antibiotics rather act by alleviating the bacterial burden.

Most studies have shown higher prevalence and incidence of rosacea in women than men and have shown a tendency for earlier diagnosis in women (48). The identification of type I IFN as key early driver in rosacea might provide one explanation. Like rosacea, most autoimmune diseases also predominantly affect females. Many immune-associated genes are encoded on the X chromosome (49, 50). While usually one of the X chromosomes in each female cell should be inactive, approximately 15% of X-linked genes escape inactivation (51). Thus, *TLR7* and *TLR8*, due to their localization on the X chromosome, might be overexpressed in females and could make them prone to type I IFN–driven inflammation and autoimmunity. This hypothesis is further supported by the finding that, in women, rosacea was significantly associated with autoimmune diseases such as type 1 diabetes mellitus, celiac disease, multiple sclerosis, and rheumatoid arthritis. In contrast, only rheumatoid arthritis was associated with rosacea among male patients (52). Interestingly, one of the only 2 genomic regions associated with all these autoimmune diseases contains TYK2 (53), a member of the Janus kinase (JAK) family playing a central role in the type I IFN signaling pathway (54). Taken together, sex-biased overexpression of the type I IFN pathway could explain an increased susceptibility for autoimmune diseases and rosacea in women.

While, in tumors, type I IFN has antiangiogenic properties (55), we found that, in the context of skin inflammation, type I IFN in fact promotes angiogenesis. However, type I IFN does not directly affect EC proliferation. Instead, similar to what has been shown for keratinocyte proliferation (56), it licences IL-22 to induce angiogenesis via upregulation of its receptor on ECs. Thus, type I IFN induces both IL-22 at the site of inflammation and concomitant upregulation of the IL-22 receptor on ECs, providing the interface between immune activation and angiogenesis. This raises the hypothesis that repetitive flare-ups with frequent type I IFN bursts and IL-22R upregulation on ECs lead to pronounced neoangiogenesis and eventually to permanent erythema, an observation that is well known from the clinic.

We have found that the different cathelicidin peptides present in rosacea show differential abilities to kill various bacterial strains and to activate the immune system. Cathelicidins, which belong to the ancestral and evolutionary conserved AMPs, are critical in the innate defence against invasion of pathogenic microbes (57), and deficiency leads to increased rates of infection. Moreover, overexpression of cathelicidins and other AMPs is critically implicated in driving inflammatory and autoimmune diseases (58, 59). Here, we show that antimicrobial potency of cathelicidin peptides and their ability to activate the immune system are clearly distinct. Normally, DNA by itself is unable to active pDCs because it cannot enter the cells spontaneously to gain access to endosomal TLR9, the pattern recognition receptor sensing DNA. It can, however, become a potent trigger of pDC activation in the presence of cathelicidin peptides (16). The differential ability of these peptides to induce type I IFN was dependent on their respective capacity to bind and condense DNA in order to enable internalization of these DNA-peptide complexes into the cells, thereby allowing activation of pDCs through TLR9. At least partially, this seems to be linked to the different charges of these peptides. The α-helical structure of cathelicidin peptides, which stabilizes interactions between the cationic peptide and the DNA helix (60) as well as the sequence and conformation of LL-37 and FR-29, are similar. However, FR-29 (+8 charge) has a higher net-positive charge than LL-37 (+6 charge) and, thus, seems to provide a more optimal peptide/DNA charge ratio in order to activate pDCs (61). The question remains, why — from an evolutionary viewpoint — various cathelicidin peptides should be present in the skin. Plenty of different cathelicidin peptides with diverging antimicrobial capacities might broaden the spectrum and improve defence against invading pathogens. However, there seems to be a fine line between control of the microbiota and the instigation of an overt immune response by commensal bacteria as seen in rosacea.

Skin infiltration by pDCs and induction of type I IFN is a common early event that plays a relevant functional role, both in disease and physiologically, in response to skin wounding or infection. Upon skin injury, transient expression of type I IFN by pDC represents an essential early trigger of skin repair (18, 29). In contrast, overproduction of type I IFN can induce several diseases and drug side effects, such as psoriasis, lupus, and anti-TNF–induced paradoxical psoriasis (21, 28, 62). The common underlying pathway raises the question how an early type I IFN overproduction by pDCs can lead to the great diversity among these entities and initiate such different clinical phenotypes and distinct immune responses. Psoriasis is mediated by Th17 cells. In lupus, the production of pathogenic autoantibodies by dysregulated B cells is a hallmark of the disease. Finally, paradoxical psoriasis represents an ongoing innate inflammation (63). One possibility is that distinct trigger factors, which drive pDC activation and type I IFN production, could shape downstream immune responses. However, type I IFN therapy of diseases such as hepatitis C, lymphoma, multiple sclerosis, or melanoma can lead to a vast spectrum of immune-mediated side effects and autoimmune adverse events (64, 65). This rather suggests that the genetic background of the patient defines the immune response and clinical phenotype induced by type I IFN. Indeed, various genetic risk variants for psoriasis are located within the IL-23/Th17 axis (66) while several susceptibility genes for lupus are associated with B cell activation and the clearance of immune complexes (67).

This also raises the question of how these diseases drive excessive pDC activation and subsequent overproduction of type I IFN. In psoriasis, Th17 cytokines IL-17 and IL-22 induce and maintain the expression of AMPs, and these processes in turn lead to increased generation of complexes with DNA. In lupus, the continuous deposition of circulating immune complexes induce ongoing pDC activation. In paradoxical psoriasis, anti-TNFs block the maturation of pDCs, which then sustain their type I IFN production. We asked what the pathomechanism underlying the enhanced pDC activation and type I IFN production in rosacea could be. One possibility is that dysbiotic bacteria upregulate TLR2, which leads to overexpression of both cathelicidin and KLK5. Subsequently, KLK5 cleaves cathelicidin into peptides with heightened type I IFN–inducing capacities as shown in this study. Another possibility is that IL-22 and other Th17 cytokines continuously upregulate AMPs. Finally, future studies will have to elucidate if specific risk gene variants exist in rosacea that contribute to a dysregulated type I IFN pathway, as it has been described for psoriasis and lupus (66, 67).

Our study identifies a link between type I IFN and neovascularization. This link might be particularly relevant for rosacea, which is characterized by neurovascular dysregulation and aberrant vasodilation to otherwise innocuous stimuli (68, 69). The characteristic flushing is limited to the central facial area. Several factors probably contribute to this unique phenotype of rosacea: the dysbiotic bacteria initiating the immune response in rosacea are associated with Demodex mites, which themselves are restricted to sebaceous area of the face. The face is particularly exposed to UV irradiation and temperature changes, known trigger factors for rosacea flare-ups. While the erythema in early rosacea is typically transient, it becomes persistent and is often accompanied by telangiectasia later in the disease course. Thus, we provide an explanation of how repetitive flare-ups eventually favor the persistent erythema by inducing type I IFN–driven and IL-22–mediated neovascularization.

In our study, we have used both in vitro and in vivo models. Each model has its limitations and, by itself, cannot encompass all aspects of the pathogenesis of rosacea. Pustule formation or more chronic manifestations of rosacea, such as sebaceous gland alterations and granulomatous changes, are not reflected by these models. However, combined with the human ex vivo data and in vitro experiments, the mouse models used in this study provide the optimal instrument to investigate the role of the innate immunity during acute flare-ups and the functional relevance of early, upstream events in the pathogenesis of rosacea.

Taken together, this study directly links dysbiosis to the induction of pathogenic skin inflammation and neoangiogenesis. Furthermore, our findings might provide a basis for the design of therapeutic strategies targeting bacteria subpopulations, pDCs and type I IFN, or IL-22 for the treatment of rosacea and prevention of acute flare-ups.

## Methods

### Human samples.

Following dermatopathological assessment, punch-biopsies were taken with informed consent from affected skin (cheeks, nose, chin, or forehead) from acute flare-ups or stabilized rosacea lesions. Samples were selected where a cutaneous lupus erythematosus diagnosis was unequivocally ruled out by immunopathological assessment. In total, 16 biopsies were obtained from 16 patients during acute flare-ups, and 16 biopsies were obtained from 16 different patients during stable disease (Supplemental Table 1). Biopsies from healthy individuals were obtained with informed consent from residual skin from aesthetic surgery of healthy individuals. Buffy coats from healthy donors were obtained from the local transfusion center and blood bank, with ethical approval from cantonal authorities.

### Mouse experimentation.

Balb/cByJ (JAX mouse strain) mice were purchased from Charles River Laboratories, and experiments were performed on age-matched animals. Intradermal injections of cathelicidin peptides were performed as previously described unless stated otherwise. Briefly, a 50 μL volume of sterile saline, or 250 μM of synthetic cathelicidin peptides (custom made from Proteogenix), was injected intradermally every 12 hours a total of 4 times, and mice were euthanized and biopsied 48 hours after first the injection. Antibodies anti-Ifnar1 (MAR1-5A3, BioXCell) and pDC depleting antibodies (BX444, BioXCell) were each injected (200 μg) 24 hours prior to intradermal cathelicidin injections. pDC depletion in Balb/c BDCA2-DTR transgenic mice was performed by injection of 120 ng of diphtheria toxin 24 hours and 4 hours prior to the first intradermal LL-37 injection. Topical antibiotic treatments were performed for 48 hours prior to intradermal injections in 2 applications, with a tailor-made ointment preparation (vaselinum album qsp 30 g, paraffinum liquidum 5 g) either containing or lacking Neomycin (105 mg), Polymyxin B (18.85 mg), and Bacitracinum (141.17 mg), purchased from International Pharmacie. For *B*. *oleronius* (ATCC, 700005) injections, the corresponding amount of bacterium inoculate for 1 × 10^4^ CFU after overnight culture was heat-killed at 55°C for 10 minutes (killing confirmed by overnight culture) and either incubated with 250 μM of LL-37, or vehicle, for 12 hours; intradermal injections were performed at 12-hour intervals.

### IHC.

For IHC of human skin, samples were fixed in 4% paraformaldehyde and paraffin-embedded. Stainings were performed using anti-CD123 (7G3, BD Pharmingen) or anti-MxA (CL143, Merck) followed by visualization using the horseradish peroxidase technique. The number of positive cells per high-magnification field was determined in relation to the number of nonstromal inflammatory cells using automated and standardized counting of round nuclei (ImageJ v1.50b, NIH).

### Immunofluorescence.

Skin biopsies (4 mm) from patients with rosacea were snap-frozen in liquid nitrogen and embedded in OCT compound prior to frozen sectioning on a microtome-cryostat. Then, 10 μm skin sections were fixed with cold methanol for 5 minutes at –20°C, permeabilized in PBS 0.012% Triton X-100 for 20 seconds at room temperature (RT), and saturated with PBS 5% normal FBS for 30 minutes at RT. Skin sections were then stained in Dako REAL Antibody Diluent (Dako) with the following fluorophore-conjugated monoclonal antibodies: BDCA-2–FITC (1/50, Miltenyi Biotec, clone AC144), CD11c-PE (1/5, BD Biosciences, clone S-HCL-3), and CD123-APC (1/20, BioLegend, clone 6H6) for 2 hours at RT. Following several washes, slides were mounted with ProLong Gold antifade mountant with DAPI (Thermo Fisher Scientific) and analyzed on a Zeiss LSM 700 confocal microscope.

### Gene expression analyses.

For quantitative PCR (qPCR), samples were homogenized by mechanical disruption using Polytron PT1200E (Kinematica) in Trizol reagent (Invitrogen), and total RNA was obtained using phenol/chloroform extraction and isopropanol, followed by ethanol precipitation. RNA was reverse transcribed using SuperScript II reverse transcriptase kit (18064014, Invitrogen), using random primers (C1181, Promega) and Oligo(dT)15 primers (C100A, Promega). qPCR was performed using “Best Coverage” TaqMan probes or SybrGreen primers (sequences indicated in Supplemental Table 2). TaqMan Gene Expression Mix (Invitrogen) or Power SybrGreen Master Mix were used for qPCR in 10 μL reaction volumes. Values are expressed as 2^–ΔΔCt^ × 10^4^ relative to the endogenous control *GAPDH/Gapdh,* and for human samples, these were normalized to healthy donor expression.

### Flow cytometry.

Biopsies of 0.6 cm in diameter were taken of the injection site, and skin was mechanically disrupted using a sterile scalpel in PBS containing 2 mM EDTA. The resulting cell suspension was stained in 0.5% FBS/PBS 2 mM EDTA using anti-B220 (RA3-6B2, BD Pharmingen), anti-Cd11c (N418, eBiosciences), anti-Cd45 (30-F11, BD Pharmingen), anti-Pdca1 (927, BioLegend), anti-Cd31 (390, eBiosciences), anti-Ki67 (SolA15, eBiosciences), and anti-BrdU (Bu20A, eBiosciences).

### Structure prediction of AMPs.

Peptide prediction was performed using the I-TASSER tool (70, 71) and visualized using Chimera 1.14 (72).

### pDC stimulations.

pDCs were isolated from buffy coats from healthy donors using the Diamond Plasmacytoid Dendritic Cell Isolation Kit II (130-097-415, Miltenyi Biotec), and cultured 5 × 10^4^ cells/200 μL in RPMI 1640 supplemented with 10% FBS and 1% penicillin/streptomycin (Thermo Fisher Scientific, 15140122). Stimulations were performed using 5 μg/mL human DNA and 10 μM or 3 μM of the indicated peptides. Live *B*. *oleronius* was added at 1 × 10^5^ CFU/mL alone or in combination with 5 μM of LL-37. IFN-α production was measured by ELISA after 24 hours of stimulation using the human pan IFN-α–specific development kit (3425-1H-6, Mabtech).

### DNA-cathelicidin peptide complex uptake.

Isolated pDCs were incubated with 3 μg/mL of Alexa Fluor 488–labeled DNA (U21650, Invitrogen) alone or in combination with 1 μM of the indicated peptide at 37°C for 3 hours in RPMI 1640 (Gibco, Thermo Fisher Scientific) supplemented with 10% FBS. For visualization by confocal microscopy, cells were cytospun onto glass microscope slides.

### DNA binding.

DNA binding and condensation activity of the cathelicidin peptides was determined using a picogreen (Invitrogen) dye fluorescence quenching technique. Peptides were added to 2 μg of purified human genomic DNA at the indicated final concentrations for 30 minutes, and picogreen dye (Quant-iT PicoGreen dsDNA Assay, Invitrogen) was used for DNA staining. Sample fluorescence was determined using 480 nm excitation and measured at 520 nm with continuous reading between 500 and 650 nm using a spectrofluorometer. When there is strong peptide-DNA binding and condensation, condensed DNA molecules are rendered inaccessible to the dye, leading to reduction in signal intensity.

### In vitro and in vivo Cramp processing.

Mouse Cramp protein (RPC419Mu01, Cloud-Clone Corp.; 100 ng) was incubated with 1.5 ng hKLK5 (1108-SE, R&D Systems) or reconstitution buffer for the indicated times, heated to 95°C for 5 minutes, and ran by Western blot alongside synthetic mCramp peptide (Proteogenix). Same-size skin punch biopsies from 7-day-old K5.*KLK5* mice and WT littermates were homogenized using Polytron PT-1200E (Kinematica) in the presence of a protease inhibitor cocktail (P8340, MilliporeSigma); immediately after sodium dodecyl sulfate (SDS) was added to a 0.4% final concentration, samples were sonicated before being heated to 95°C for 5 minutes, and they ran by Western blot alongside mCramp peptide and protein. Actin was probed as loading control.

### Bacterial killing.

Bacterial strains *B. oleronius* (ATCC, 700005), *S. epidermidis* (ATCC, 14990), *C. acnes* (ATCC, 6919), *Klebsiella pneumoniae* (ATCC, 10031), *Pseudomonas aeruginosa* (ATCC, 27853), and *E. coli* (ATCC, 700973) were prepared at the indicated working concentrations and incubated with the indicated peptide at working concentrations ranging from 0.2 to 10 μM for 5 hours in PBS. They were plated, and CFU were counted after overnight incubation at 37°C — except for *C*. *acnes,* which was incubated under anaerobic conditions. Values are expressed as percentage of survival.

### NHEK culture and stimulation.

Commercially available primary normal human epidermal keratinocytes (NHEK) cells (Promocell) were cultured under serum-free conditions, supplemented with Keratinocyte Growth supplements (Promocell) as per the manufacturer’s instructions. Live *B*. *oleronius* and *C*. *acnes* were incubated with NHEK cells at 1 × 10^3^ CFU (calculated from overnight plating) for 24 hours. After 24-hour stimulation, RNA was extracted from cells by phenol-chloroform extraction, and qPCR was performed for *KLK5* (Hs01548153_m1).

### HUVEC culture, proliferation, and survival.

HUVECs (ATCC, CRL-1730) were cultured in growth media (EGM-2 Endothelial Cell Growth Medium-2 BulletKit, Lonza) containing growth factors IGF1, FGF2, EGF, and VEGFA according to the manufacturer’s instructions, unless otherwise stated. Cells (5 × 10^3^ per 96-well) were stimulated with 1,000 U/mL human IFN-α (1100-1, PBL Assay Science), and/or 100 ng/mL IL-22 (200-22, PeproTech) for 8 hours for gene expression analyses or for 5 days for survival and proliferation analyses. For survival experiments, cells were stained with SYTO 13/SytoX ORANGE (S34854, S11368, Invitrogen) and analyzed by flow cytometry. Live cells were counted as total Syto single-positive cells per condition. For proliferation experiments, cells were fixed, permeabilized (FIX & PERM Cell Fixation & Permeabilization Kit, Thermo Fisher Scientific), stained for KI67, and analyzed by flow cytometry.

### Mouse EC proliferation.

To determine in vivo mouse EC proliferation, mice were treated with the indicated antibody prior to induction of cathelicidin-mediated skin inflammation, as previously, and injected i.p. with 2.5 mg of BrdU (19-160, Merck) in 200 μL saline 24 hours prior to the end of the injection regimen. Biopsies measuring 0.28 cm^2^ of the injection sites were taken; cell suspensions were prepared as described previously, stained for Cd45 and Cd31, fixed and permeabilized as described in the previous section, and stained for Ki67 and BrdU. The percentage of proliferating cells was determined as single-positive Ki67 (initiating proliferation), Ki67-BrdU double-positive cells (sustained proliferation), and single-positive BrdU (previously proliferating).

### Videocapillaroscopy and human dermoscopy.

For mouse skin videocapillaroscopy, mice were anesthetized with a ketamine-xylazine cocktail (100 mg/kg and 10 mg/kg, respectively) to reduce mobility and slow respiration for more stable imaging. Injection sites and adjacent areas were delicately ointed with an immersion oil, and microvessels were visualized using a clinical nailfold videocapillaroscope Xport Technology capXview. Images were acquired with the software capXview HD2 version 5.0. Microvessel numbers were determined by manual counting from images of injection sites and adjacent areas using ImageJ v.1.8.0_191. Rosacea microvessels were visualized with a FotoFinder medicam 800 HD.

### Statistics.

Two-tailed unpaired Student’s *t* test or 1-way ANOVA with Dunnett’s multiple comparisons were used as appropriate, unless otherwise stated in the figure legend. Statistical analyses were performed with GraphPad Software (version 8.2.1). Significance threshold was set to *P* < 0.05.

*Study approval* Studies with human biopsies were approved by the local IRB of Lausanne, Switzerland (ethical approval no. 265/12) and Dusseldorf, Germany (ethical approval no. 2048). Animal experiments were performed according to institutional guidelines and the Swiss Federal Animal Protection Act and cantonal laws on animal protection. Consent was received from the Swiss Federal Food Safety and Veterinary Office (ethical approval no. VD3299). Buffy coats from healthy donors were obtained from the local transfusion center and blood bank, with ethical approval from cantonal authorities.

## Author contributions

CC formulated the hypotheses and designed and supervised the study. CC and AM performed and supervised experiments, interpreted data, and wrote the manuscript. OD provided critical expertise on the in vivo model. HCH, SM, and BH provided human rosacea biopsies, performed expression profiling of samples, and contributed critical appraisal of the manuscript. OD, JDD, and MG provided invaluable appraisal of the manuscript and shaped the hypothesis of the work. YW and AH provided transgenic mouse models, samples, shaped the hypothesis of the work, and contributed critical appraisal of the manuscript. JC and FM performed experiments and immunostainings for revisions. LM provided expertise on videocapillaroscopy and angiogenesis, and contributed critical appraisal of the manuscript.

## Supplementary Material

Supplemental data

## Version 1 01/12/2023

In-Press Preview

## Version 2 02/22/2023

Electronic publication
