# Supplementary material for: Type I IFNs link skin-associated dysbiotic commensal bacteria to pathogenic inflammation and angiogenesis in rosacea
Source: JCI Insight. 2023 Feb 22;8(4):e151846. doi: 10.1172/jci.insight.151846 (PMC9977509; doi:10.1172/jci.insight.151846)
Supplement: Supplemental data [file jciinsight-8-151846-s222.pdf]

Supplementary Figure 1

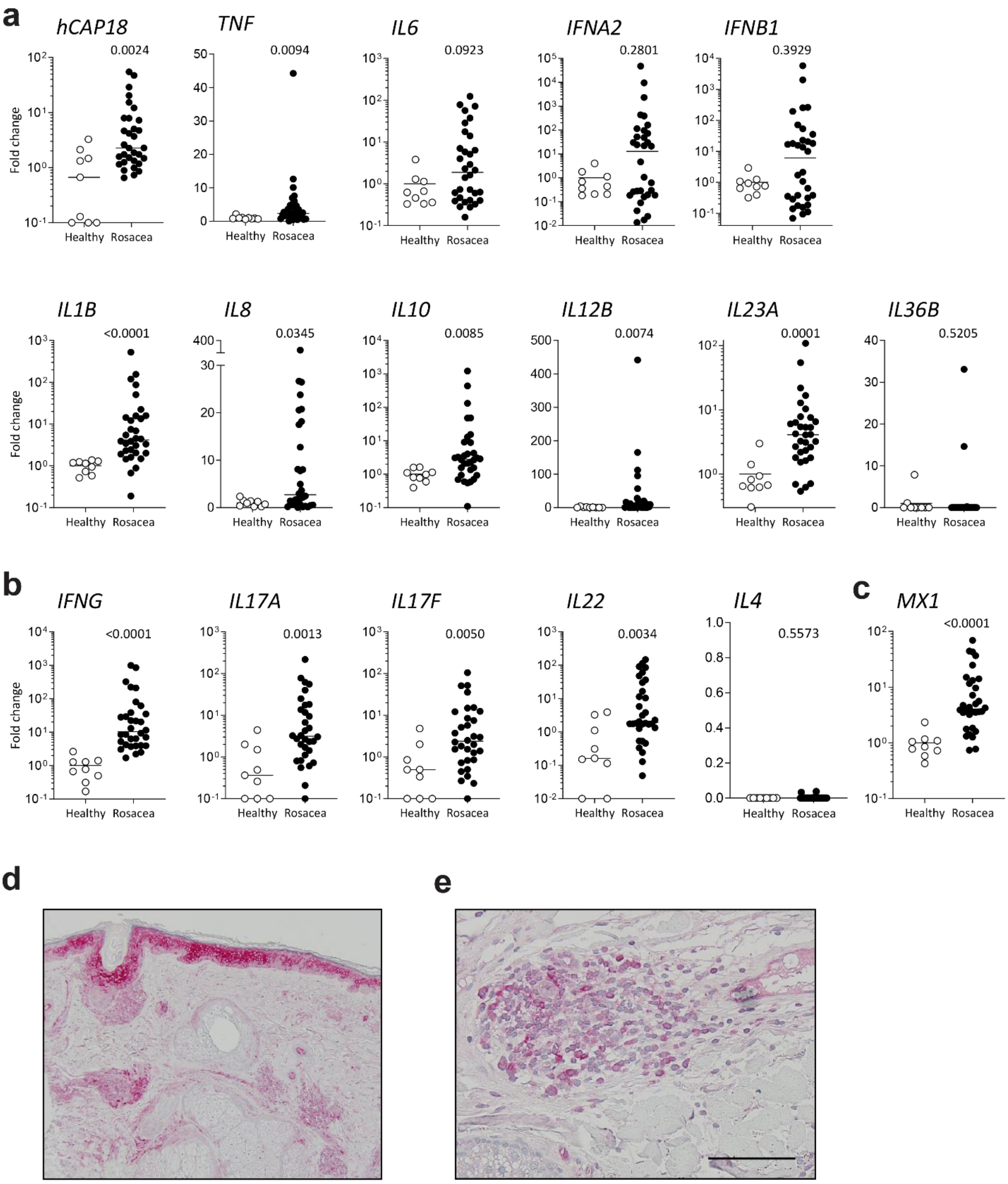

Fig. S1: Inflammatory cytokine profile in lesions of rosacea, and MxA staining (**a**) Gene expression of innate and (**b**) adaptive inflammatory genes from biopsies of lesions of rosacea (**black**, n=32) as compared to healthy skin (**white**, n=9). (**c**) *MX1* expression comparison between healthy skin and rosacea lesions. P-values of two-tailed Mann-Whitney nonparametric unpaired t-test are depicted. (**d**) MxA staining of rosacea in whole skin, and (**e**) of dermal infiltrate. Scale bar represents 100µm.

Supplementary Figure 2

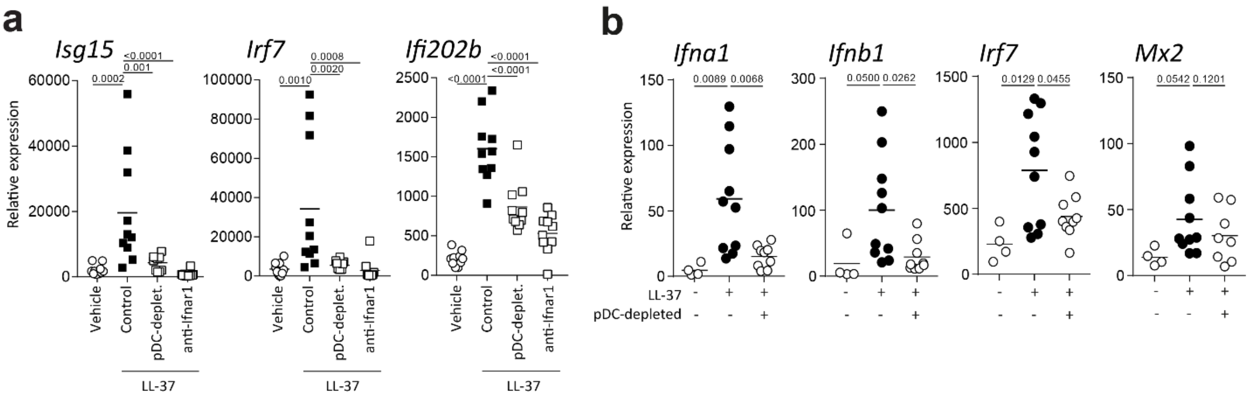

Fig. S2: Type I Interferon and response genes require pDCs *in vivo*. Gene expression from biopsies following LL-37 intradermal injection in mice (**a**) depleted of pDCs via antibody-mediated targeting or blockaded of type I interferon signalling, (**b**) or depleted of pDCs via diptheria toxin injection in BDCA2-DTR transgenic mice. Multiplicity adjusted P-values of one-way ANOVA are depicted.

Supplementary figure 3

a

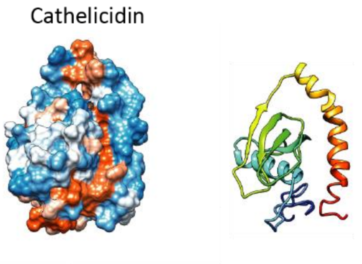

b

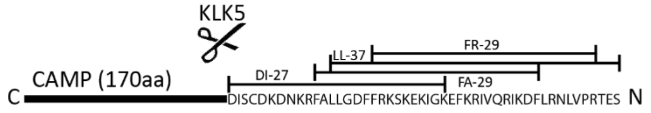

c

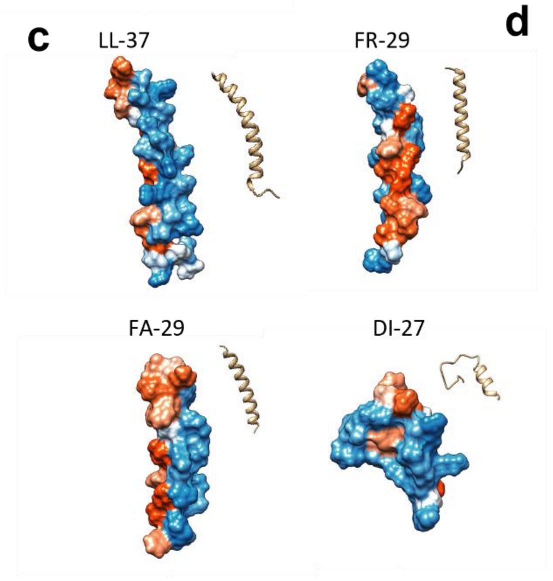

d

Peptide characteristics

	Charge, %	Helical, %	Solvent accesible area
LL-37	+6	75.68	4420.79
FR-29	+8	82.76	3324.11
FA-29	+5	82.76	3205.09
DI-27	+2.9	29.62	2691.82

Fig. S3: Cathelicidin peptides described in rosacea. (**a**) Cathelicidin structure prediction with hydrophobicity colouring of residues depicted (phobic to philic: red to blue). Ribbon representation coloured N- (blue) to C-terminus (red). (**b**) Cathelicidin Antimicrobial Peptide (CAMP) processing by Kallikrein 5 (KLK5) yields different sized antimicrobial peptides DI-27, FA-29, LL-37, and FR-29 as described elsewhere.(13) (**c**) Structure prediction and ribbon representation of cathelicidin antimicrobial peptides, and (**d**) their accompanying characteristics. Predictions obtained using I-TASSER.(70, 71)

Supplementary figure 4

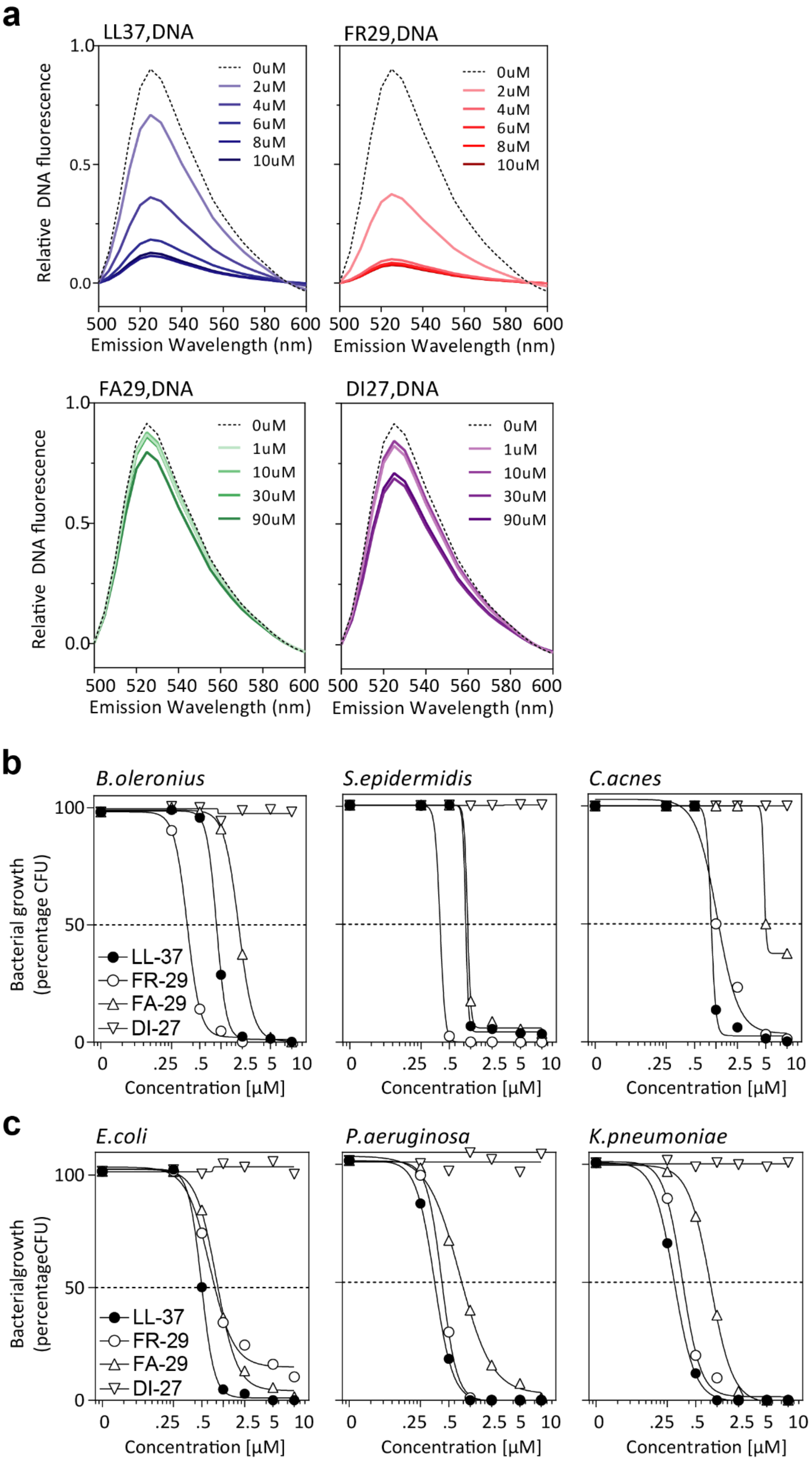

Fig. S4: Differential DNA binding capacities and bacterial killing of cathelicidin antimicrobial peptides described in rosacea skin lesions. (**a**) Human DNA (2µg) was incubated with varying molar concentrations of the indicated cathelicidin peptides, and unbound free DNA was quantified by picogreen dye. (**b**) Skin- and (**c**) gut- and respiratory tract-associated bacterial killing assay by indicated cathelicidins. Bacteria CFU counts were determined after 18h culture in their appropriate culture conditions and the corresponding 10^5 CFUs were incubated with the indicated peptides at the indicated concentration for 2h, followed by plating and culturing for 18h. CFUs were counted and calculated as relative percentage of control cultures.

Supplementary Figure 5

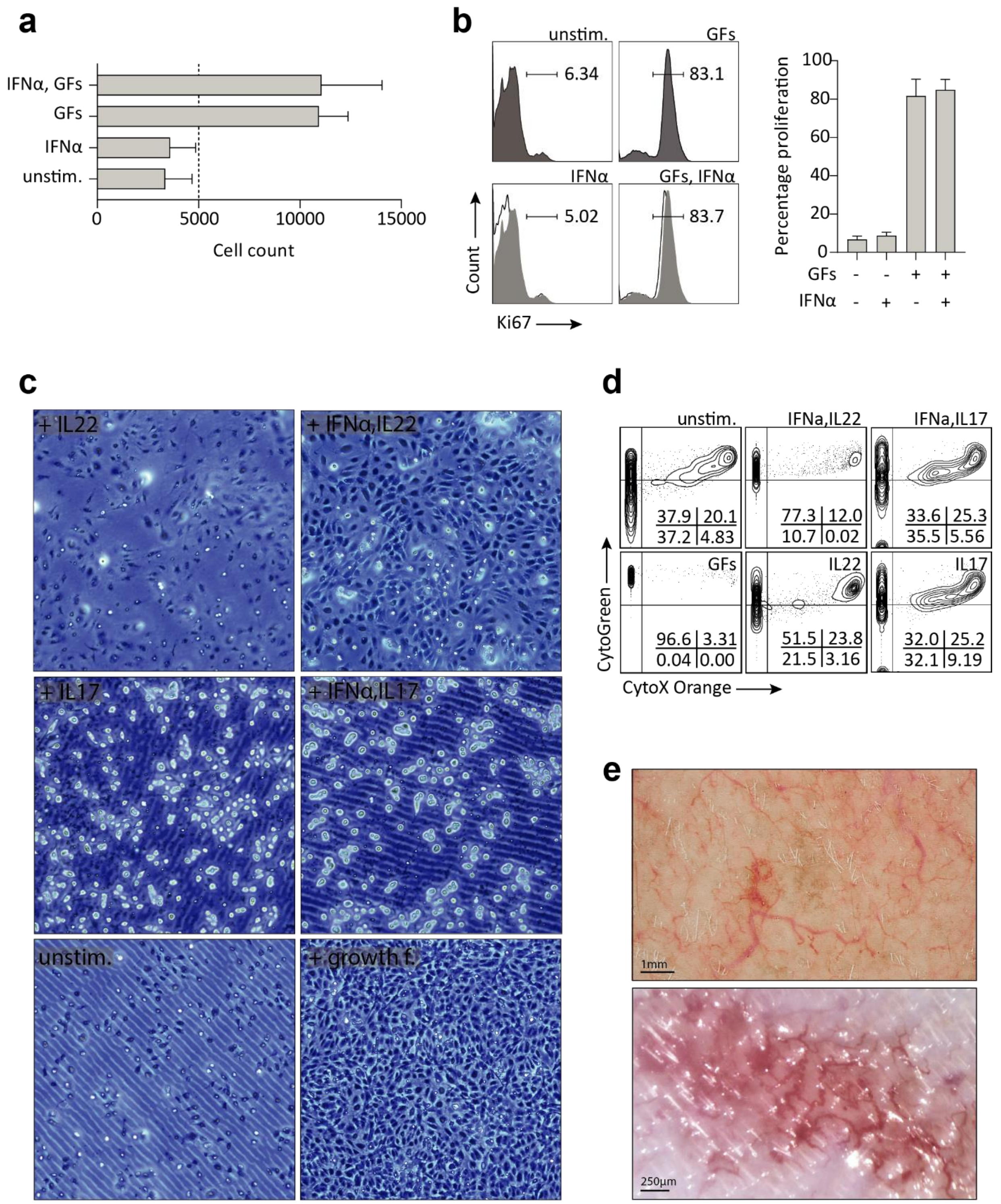

Fig. S5: IFNα does not directly induce Huvec proliferation or survival, despite participating in the observed angiogenesis *in vivo*. (**a**) Cell survival of Huvec stimulated with or without IFNα, in the presence or absence of growth factors (GFs). (**b**) Huvec proliferation determined by KI67 staining of cells stimulated as in (a). (**c**) Huvec stimulated with indicated cytokines, left unstimulated, or with the growth factor cocktail, and visualised by light microscopy. (**d**) Huvec were stimulated with the indicated conditions as in (c) and survival assessed by SYTO®13 Green and SYTOX® Orange staining. (**e**) Representative images of rosacea skin taken with fotofinder (upper panel) and videocapillaroscopy images of LL-37 injection site (bottom panel).

Supplementary figure 6

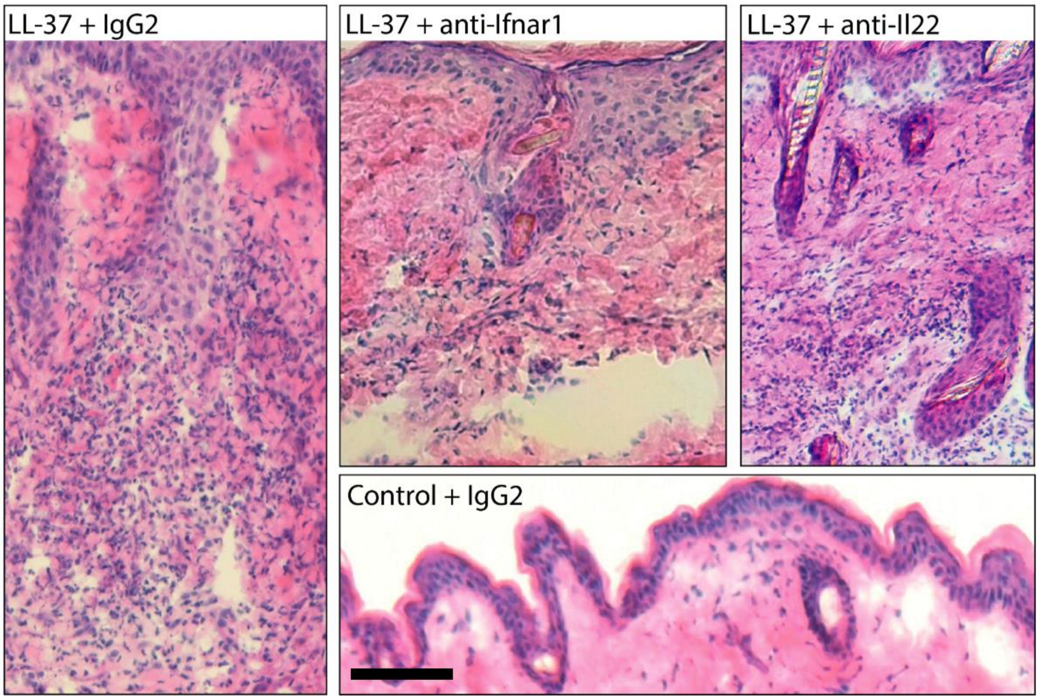

Fig. S6: H&E staining of selected sections from mice injected intradermally either with LL-37 or Saline, and which received either an anti-mouse Ifnar1 or Il22, or an IgG2 control. Dotted lines highlight lumens of dilated blood vessels. Scale bar represents 50μm.

Supplementary Table 1: Patient characteristics

Phase	Rosacea subtype (%)			Age at biopsy	Gender (%)	
	PPP	ETR	Ph	(Median ± SD)	F	M
Stable	83.33	38.88	5.55	57 ± 9.94, n=16	50	50
Flare	50	68.75	0	48.5 ± 11.57, n=16	50	50

ETR: Erythematotelangiectatic, PPP: Papulopustular, Ph: Phymatous

Supplementary Table 2: Sequences of SybrGreen® primers used

Gene	Forward (5'-3')	Reverse (5'-3')
GAPDH	ACG CAT TTG GTC GTA TTG GG	TGA TTT TGG AGG GAT CTC GC
IL6	GGTACA TCC TCG ACG GCA TCT	GTG CCT CTT TGC TGC TTT CAC
IFNG	ACG TCT GCA TCG TTT TGG GTT	GTT CCA TTA TCC GCT ACA TCT GAA
IL10	TCC TGA CTG GGG TGA GGG CC	GGC AGG TTG CCT GGG AAG TGG
TNF	AGT GAT CGG CCC CCA GAG GG	CAC GCC ATT GGC CAG GAG GG
IL36B	TTC TGT GCA GAA ATT CAG GGC	TCC CTA TGT TAT CTT GGG AGC C
IL1B	CAC GAT GCA CCT GTA CGA TCA	AGA CAT CAC CAA GCT TTT TTG CT
MX1	AGA GAA GGT GAG AAG CTG ATC	TTC TTC CAG CTC CTT CTC TCT G
IFNA2	TCC TGC TTG AAG GAC AGA CA	TTT CAG CCT TTT GGA ACT GG
IFNB1	CGA CAC TGT TCG TGT TGT CA	GAA GCA CAA CAG GAG AGC AA
